# Triazolo[4,5*-d*]pyrimidines as Validated General Control Nonderepressible 2 (GCN2) Protein Kinase Inhibitors Reduce Growth of Leukemia Cells

**DOI:** 10.1016/j.csbj.2018.09.003

**Published:** 2018-09-28

**Authors:** Lea Lough, Dan Sherman, Manuel Becerra-Flores, Deepika Vasudevan, Olga Lavinda, Eric Ni, Hong Wang, Hyung Don Ryoo, Raoul Tibes, Timothy Cardozo

**Affiliations:** Department of Biochemistry and Molecular Pharmacology, New York University School of Medicine, New York, NY 10016, USA; Department of Laura & Isaac Perlmutter Cancer Center, New York University School of Medicine, New York, NY 10016, USA

## Abstract

Cellular stress signals activate adaptive signaling pathways of the mammalian integrated stress response (ISR), of which the unfolded protein response (UPR) is a subset. These pathways converge at the phosporylation of eIF2α. Drug-like, potent and selective chemical inhibitors (valid chemical probes) targeting major ISR kinases have been previously identified, with the exception of GCN2. We synthesized and evaluated a series of GCN2 inhibitors based on a triazolo[4,5*-d*]pyrimidine scaffold. Several compounds potently inhibited GCN2 *in vitro* and displayed good selectivity over the related kinases PERK, HRI, and IRE1. The compounds inhibited phosporylation of eIF2α in HEK293T cells with an IC_50_ < 150 nM, validating them as chemical probes for cellular studies. These probes were screened against the National Cancer Institute NCI-60 human cancer cell line panel. Uniform growth inhibition was observed in the leukemia group of cell lines. Growth inhibition in the most sensitive cell lines coincided with high GCN2 mRNA expression levels. Oncomine analysis revealed high GCN2 expression accompanied by lower asparagine synthetase (ASNS) expression in patient-derived acute lymphoblastic leukemias with B-Cell origins (B-ALL) as well. Notably, asparaginase, which depletes amino acids and triggers GCN2 activity, is a licensed, first-line B-ALL treatment. Thus, we hypothesize that leukemias exhibiting high GCN2 expression and low ASNS expression may be susceptible to pharmacologic GCN2 inhibition.

## Graphical Abstract

**Figure:**
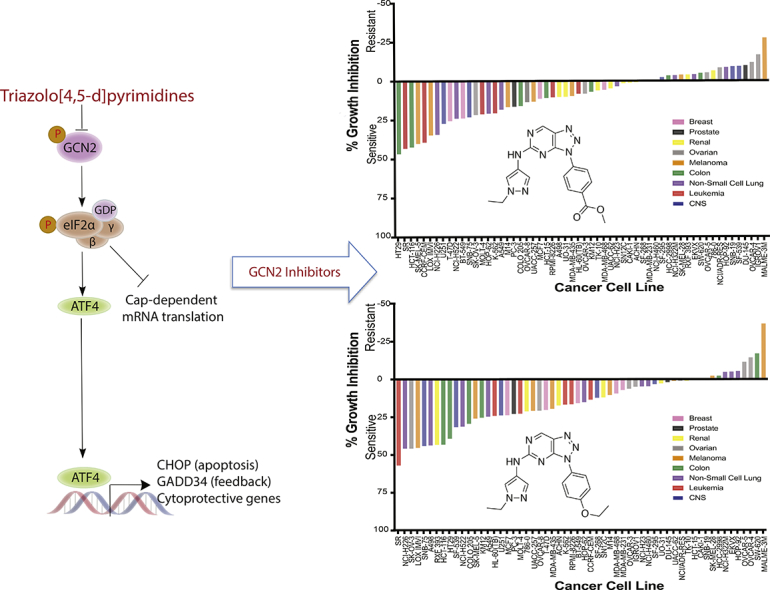

## 1 Introduction

**Fig. 1:**
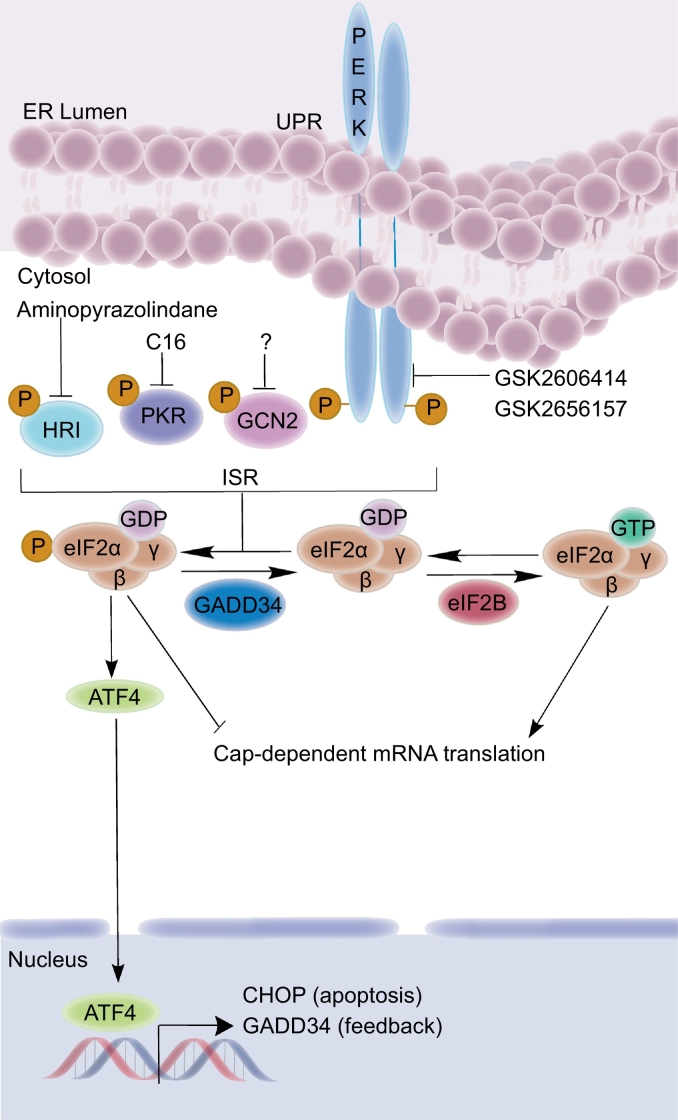
ISR signaling pathway and known ISR kinase inhibitors.

The integrated stress response (ISR) is a signaling pathway in eukaryotic cells that responds to different physiological and pathophysiological stress signals. The function of the ISR is to restore cellular homeostasis by attenuating global translation and by upregulating cytoprotective genes. However, if homeostasis is not restored, or if stress persists, then apoptosis is initiated. The ISR is regulated by four kinases that become active in response to different stressors: PKR-like ER kinase (PERK) responds to accumulation of unfolded or misfolded proteins in the endoplasmic reticulum (ER) [1] and is one of three proteins (PERK, activating transcription factor 6 (ATF6) [2], and inositol-requiring enzyme-1 (IRE1) [3,4]) that activates the unfolded protein response (UPR) upon ER stress; general control nonderepressible 2 (GCN2) responds to amino acid starvation [5,6] and UV light [7,8]; double-stranded RNA-dependent protein kinase (PKR) responds to viral infection (double stranded RNA); and heme-regulated eIF2α kinase (HRI) responds to heme deficiencies [[9], [10], [11]]. All these kinases in turn converge in activating the ISR by phosphorylating the eukaryotic translation initiation factor 2 alpha (eIF2α) [10]. This phosphorylation event attenuates cap-dependent mRNA translation (thereby reducing protein load stress in the ER) and amplifies translation of mRNAs with upstream open reading frames (uORFs) in their 5′-UTRs, including the activating transcription factor 4 (ATF4) (Fig. 1) [9,12]. ATF4 in turn controls the expression of cytoprotective, pro-apoptotic, and negative feedback genes. Specifically, DNA damage-inducible 34 (GADD34) aids in the dephosphorylation of eIF2α to return mammalian cells to normal translation following stress, while a build up of C/EBP-homologous protein (CHOP) triggers apoptosis (Fig. 1) [13,14].

ISR markers have been identified in numerous cancer cell lines and human tumor tissues [[15], [16], [17]]. These data have implicated different ISR kinases in tumor development and progression wherein tumor cells are susceptible to microenvironment insults, such as nutrient deprivation and hypoxic conditions. For instance, PKR is overexpressed and constitutively activated in acute leukemia cells and breast cancer cells [18,19], while GCN2 and PERK are essential for efficient tumorigenesis and tumor progression [20]. The role of HRI in cancer is still unclear. Interestingly, GCN2 appears to be able to compensate for loss of PERK by phosphorylating eIF2α [[20], [21], [22], [23]].

GCN2 activation in response to amino acid deprivation is a mechanism by which tumor cells cope with nutrient stress and as a result can promote tumor angiogenesis and growth [24]. Many tumors lack enzymatic machinery to synthesize non-essential amino acids [25]. For instance, leukemia cells lack the ability to synthesize asparagine [26]. Thus, asparaginase, which functions by depletion of asparagine and glutamine, is a first line of treatment for B-Cell-derived acute lymphoblastic leukemia (B-ALL) [27]. Since GCN2 is capable of reversing chemotherapeutic amino acid deprivation, it is a promising drug target. Indeed, GCN2 has been shown to alleviate asparaginase-induced stress in normal lymphocytes *in vivo* [28]. The role of GCN2 in other cancers has also been investigated; for example, tumor xenograft studies of head and neck squamous cell carcinoma (HNSCC), or fibrosarcoma (HT1080) cell lines with GCN2 deletions prevented tumor growth and survival [17,24]. Additionally, in the case of BRAF-mutant melanoma and colorectal cancer lines treated with vemurafenib, GCN2 adopted a cytoprotective role: both cancers regained sensitivity to the drug after shRNA knockdown of GCN2, making this kinase a possible target to combat vemurafenib resistance [29]. These studies illustrate both the promise and the concerns surrounding GCN2 as a drug target in cancer.

High quality small molecule probes specific to each of the ISR kinases, except GCN2 have been reported. Inhibitors of PERK (GSK2606414 and GSK2656157) [30], PKR (C16) [31], and HRI (aminopyrazolindane) [32] are known (Fig. 1). Notably, the diversity of structure-activity relationships (SAR) exhibited by the PERK, PKR, and HRI inhibitors visualized in complex with their target kinases may inform the inhibitory conformation of the GCN2 kinase domain, if they could be compared to a GCN2 inhibitor. There are currently no selective and potent inhibitors of GCN2 reported in the peer-reviewed literature, although several GCN2 inhibitors were disclosed in the patent database with unverified bioactivity [33]. Recently, Nakamura et al. reported on a set of GCN2 inhibitors that showed no growth inhibition when tested alone against different cancer cell lines [34]. Accordingly, we prepare a series of compounds containing a triazolo[4,5-*d*]pyrimidine core with the goal to validate their potency and selectivity for GCN2 kinase. Compounds **1** and **2** were further used to test the emerging hypothesis in the field that GCN2 is a valid drug target for B-ALL.

## 2 Results and Discussion

### 2.1 Chemistry

**Fig. 2:**
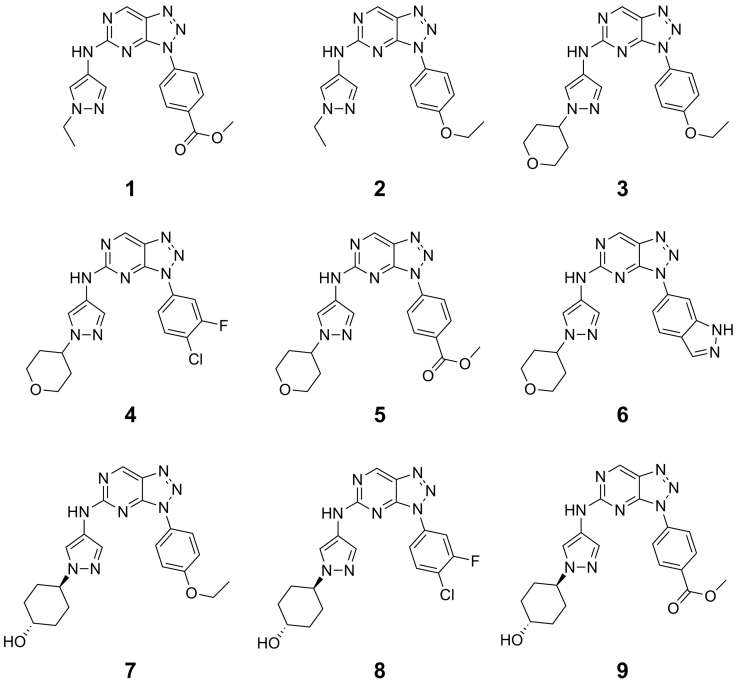
Triazolo[4,5-*d*]pyrimidines synthesized as GCN2 inhibitors.

We synthesized a set of the triazolo[4,5-*d*]pyrimidines [33], which were disclosed in a non-peer-reviewed patent application, in order to identify a tool compound that could serve as a benchmark for cellular based screening of GCN2 inhibition. Only unconfirmed relative biochemical GCN2 potency was disclosed in the patent. We therefore synthesized a set of compounds that were both decorated with the most frequently used sub-structural motifs and were suggested by the patent to have sub-micromolar activity in enzymes and cells (Fig. 2**, 1–9** & Supplementary Material S1).

### 2.2 *In-Vitro* Testing

**Fig. 3:**
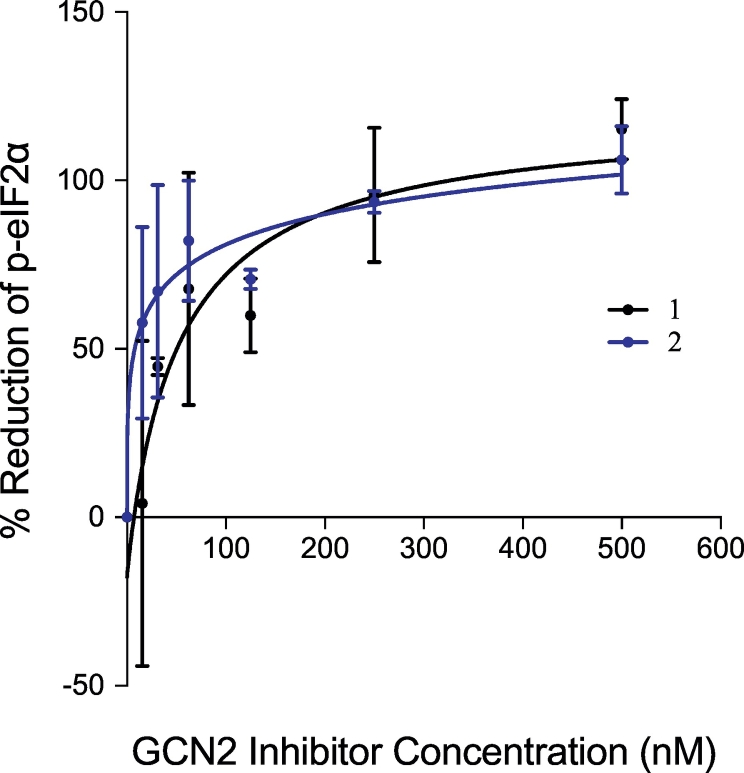
*In-vitro* cell-based IC_50_ curves of p-eIF2α reduction using compounds **1** and **2**.

Compounds **1**–**9** were screened in a radiometric [32]P-ATP kinase assay against human GCN2 and other kinases involved in the UPR and ISR pathways (Table 1). The compounds tested inhibited GCN2 with good potency (18.6 nM to 46.4 nM) and most of these compounds showed selectivity towards GCN2 over PERK, HRI, and IRE1 (Table 1). Strong cross-inhibition was observed between GCN2 and PKR, revealing this series to be potential dual-action kinase inhibitors and have potential selectivity issues. Compound **2** was additionally screened against a standard broad kinase panel and no significant kinase inhibition was seen (Supplementary Material S2). Compounds **1**–**9** were potent, permeable, but poorly soluble (Table 1). The most soluble of the potent tool compounds (**1** and **2**) were selected for *in vitro* cell-based assays where the phosphorylation of eIF2α (p-eIF2α) was used as a readout for GCN2 inhibition. Both compounds had similar mechanistic IC_50_ values of 52.6 nM (**1**) and 138.4 nM (**2**) (Fig. 3).

**Table 1:** Inhibitory potencies, solubility, permeability and structures of triazolo[4,5-*d*]pyrimidine derivatives.

Compound		GCN2 IC_50_	PKR IC_50_	% Enzyme inhibition at 10 μM				Solubility at pH 7.4	PAMPA^a^
ID	Structure	(nM)	(nM)	PERK	PKR	HRI	IRE1	(μM)	10^–^^6^ cm/s
GSK2656157		>10,000		100					
1		47. 6	119.3	24%	93%	14%	37%	0.65	16.0
2		18.6	39.9	<10%	99%	<10%	20%	0.31	15.14
3		44.5		25%					
4		25.7		<10%	96%	<10%	18%	0.05	2.19
5		17.2		<10%	96%	<10%	22%	0.20	10.47
6		20.5		35%					
7		22.4		24%					
8		21.1		<10%	98%	<10%	15%	0.10	4.68
9		46.4		14%	95%	<10%	24%	0.17	2.29

All data are means of two independent experiments.

a Compounds with PAMPA <10 × 10^–^^6^ cm/s are classified to have low permeability and compounds with a PAMPA >10 × 10^–^^6^ cm/s are classified as having high permeability.

**Fig. 4:**
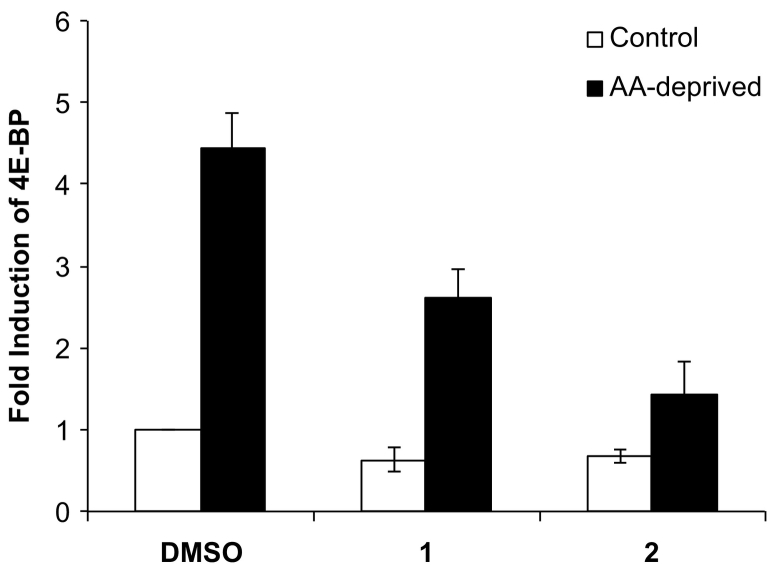
qRT-PCR of 4E-BP mRNA. AA, amino acid.

It is known that under normal nutrient conditions the cap-dependent translational inhibitor 4E-BP is not expressed [35]. However stress such as nutrient deprivation is known to trigger the transcriptional induction of 4E-BP. It has recently been shown that GCN2 and its downstream transcription factor, ATF4, mediate 4E-BP induction under amino acid deprivation in *Drosophila*, S2 cells [36]. To further investigate the downstream effects of **1** and **2** on the amino acid deprivation GCN2 − ATF4 − 4E-BP pathway [36], the levels of 4E-BP mRNA transcript were monitored. We performed qRT-PCR on *Drosophila* S2 cells. As shown in Fig. 4, the relative 4E-BP mRNA levels in cells treated with **1** or **2** under amino acid deprived conditions exhibited decreased 4E-BP transcript levels when compared to the DMSO control, suggesting selective inhibition of downstream GCN2 signaling in cells upon amino acid deprivation.

### 2.3 GCN2 3D Homology Model

The inhibitor-bound crystallographic structure of the PERK kinase (PDB: 4X7K) [37] was used as a template for building a GCN2 kinase domain homology model. The structural relationship between this template and the GCN2 kinase domain sequence was highly significant (pP = 10^–20.9^) [38], and the template structure was of high resolution (1.8 Å) and contained a small molecule inhibitor bound to the active site, which are highly desirable features of a homology model template. The bound inhibitor, 4-{2-amino-3-[5-fluoro-2-(methylamino)quinazolin-6-yl]-4-methylbenzoyl}-1-methyl-2,5-diphenyl-1,2-dihydro-3H-pyrazol-3-one, previously exhibited dual biochemical inhibition of PERK and GCN2 kinase activity with IC_50_ values of 4 nM and 36 nM, respectively [37]. Thus, we hypothesized that this inhibitor pose could capture, partly or wholly, the mode of GCN2 inhibition of **1** and **2.** Specifically, several 3D GCN2 homology models were generated and screened based on their ability to discern between known GCN2 inhibitors and non-inhibitor decoys from experimental data, the WO 2013110309 patent application, and the ChEMBL database [33,37,39]. GCN2 models that passed the screening criteria were used for docking of **1** and **2** and screened based on their ability to discern between known GCN2 inhibitors and non-inhibitor decoys from experimental data, the WO 2013110309 patent application, and the ChEMBL database [33,37,39].

### 2.4 In Silico Docking Studies

**Fig. 5:**
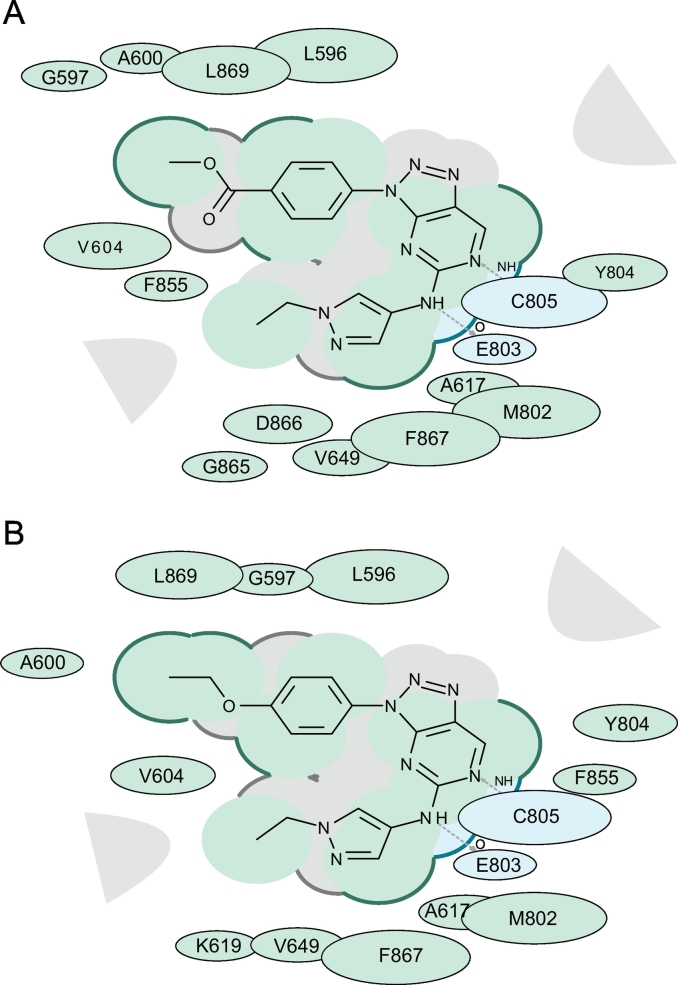
2D docked poses of **1** and **2** within the active site of GCN2. A. 2D docked pose of **1**. B. 2D docked pose of **2**. Figure demonstrates hydrophobic regions (green), hydrogen bond acceptor (blue) and hydrogen bonds (grey dashed arrows). Accessible surface for large areas (grey parabolas) and accessible surface (broken thick line around ligand shape indicates) are also shown. The size of residue ellipse represents the strength of the contact and distance between residue and ligand represents proximity.

To identify the exact mode of binding of **1** and **2** with GCN2, we used computational molecular docking of **1** and **2** *in silico* to the ATP-binding site of the N-terminal kinase domain of the generated GCN2 3D models. Docking of **1** and **2** resulted in favorable docking scores of −39.75 and −36.96 (typical threshold of significance is −32), respectively, indicating the high likelihood of these compounds to dock to the observed GCN2 site in the observed orientaton [40]. The docking results for **1** and **2** are seen in Fig. 5. In multiple docking runs, **1** and **2** consistently adapted a U-shaped binding mode that placed the pyrazole moiety facing the solvent within the ATP-binding site pocket. Notably, both **1** and **2** formed bidentate hydrogen bonds with the backbone of the hinge at glutamic acid 803 (E803) and cysteine 805 (C805) (Fig. 5).

### 2.5 NCI-60 Human Tumor Cell Line Screen

#### 2.5.1 Cytotoxic activities of compound **1** and compound **2**

**Fig. 6:**
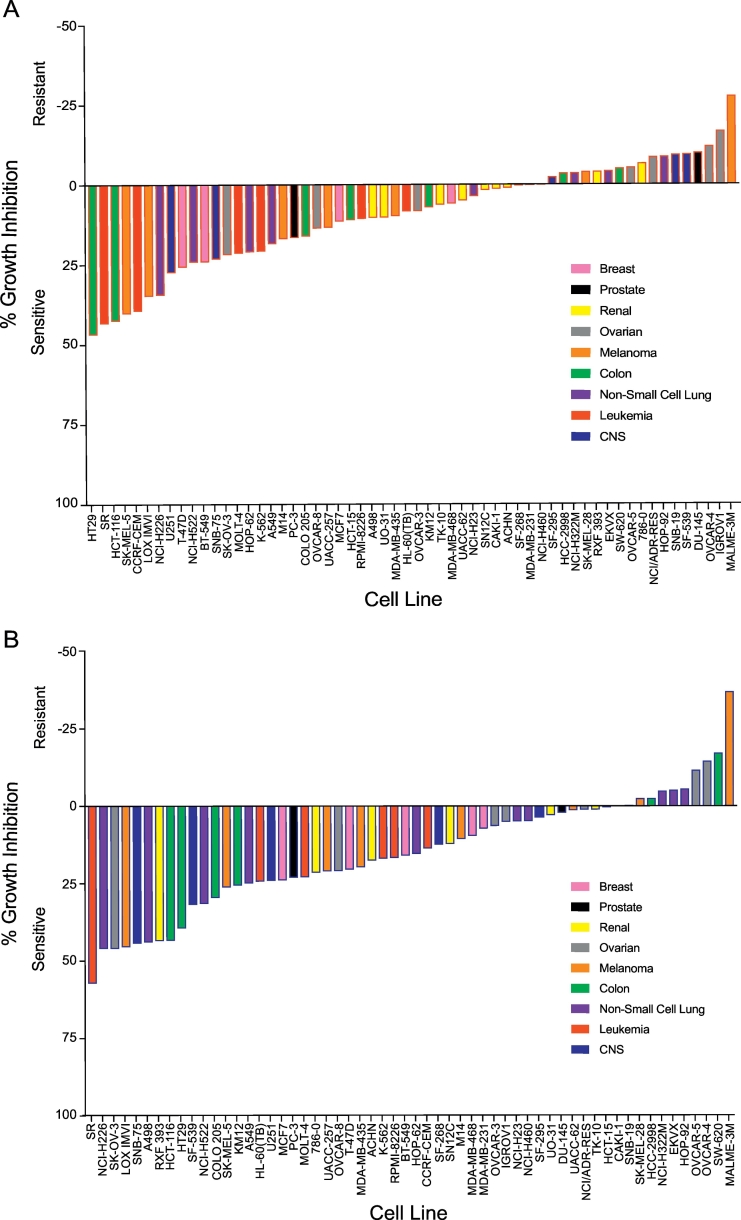
Percent growth inhibition of **1** and **2** from the NCI-60 one-dose scree. A. Percent growth inhibition of **1**. B Percent growth inhibition of **2**.

Compounds **1** and **2** were submitted to the National Cancer Institute NCI-60 Tumor Cell Screening Program (NCI number 800,700 and 800,701, respectively) for evaluation against the 60 cell line NCI panel at a single dose of 10 μM. Both **1** and **2** displayed cytotoxic activity against cells within the different types of cancers tested (Fig. 6 & Supplementary Material S3). Leukemia and breast cancer cell lines comprised the only cancer groups that were uniformly sensitive to both **1** and **2,** with the greatest sensitivity seen in the leukemia SR cell line. The highest activity for **1** was 47% growth inhibition for the colon cancer cell line, HT29, followed by 43.4% growth inhibition for the leukemia cell line, SR, and a 42.7% growth inhibition for the colon cancer cell line, HCT-116 (Fig. 6A). For **2**, the highest growth inhibition was 57.5% for the leukemia cancer cell line, SR, followed by a 46.3% growth inhibition for the non-small cell lung cancer cell line, NCI-H226, and a growth inhibition of 46.3% for the ovarian cancer cell line, SK-OV-3 (Fig. 6B). The resistant cell lines exhibited an increase in cell growth with the greatest increase seen in the melanoma cell line, MALME-3 M, by 27.7% and 37.2% with **1** and **2**, respectively. Notably, the major exceptions were seen in seven cell lines (DU-145, RXF 393, 786-0, NCI/ADR-RES, IGROV1, SNB-19, and SF-539) where both **1** and **2** exhibited contradictory growth trends.

#### 2.5.2 NCI COMPARE Analysis

**Fig. 7:**
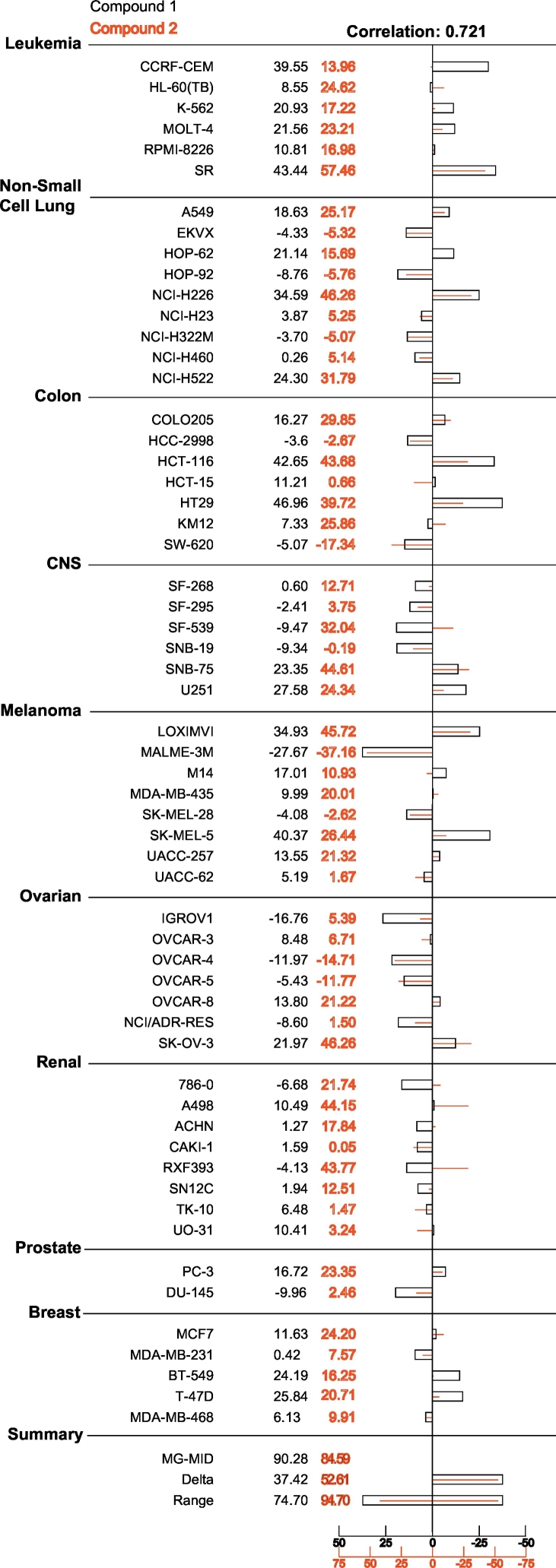
Activity correlation of **1** and **2**. Overlay of **1** (black) and **2** (red). Values were subtracted from 100 to show percent growth inhibition of cell line. Zero on the X-axis indicates the mean growth percentage of corresponding cell lines. Growth percentage of each cell line relative to the mean is represented horizontally on axis. Right side indicates more sensitivity and left side indicates less sensitivity.

The NCI COMPARE measures the degree of correlation between the pattern of cell line sensitivity between any two compounds. This algorithm was used to compare the inhibition profile for both **1** and **2** to prior NCI-60 cell panel experiments. The results revealed that both compounds share similar activity with a Pearson's correlation coefficient (PCC) of 0.721 (Fig. 7).

NCI COMPARE can also compare tested compounds with known drugs profiled against the NCI-60 panel in the NCI Standard Agents database to identify reference compounds with a similar mechanism of action to **1** and **2**. Maytansine [41], a microtubule inhibitor, was identified as the top reference compound for both **1** and **2** with weak GI_50_ PCC values of 0.519 and 0.554, respectively. No other compound had a correlation coefficient above 0.5 (Table 2), indicating that **1** and **2** have novel bioactivity, at least with respect to the Standard Agents database repository of NCI-60-tested compounds.

**Table 2:** Top NCI Standard Agents with similar activity profiles to 1 and 2.

Compound	Vector correlation	PCC	Mechanism of action	Cell lines
1	Maytansine^a^	0.554	Bind to tubulin & inhibit microtubule assembly [41]	41
1	Batracylin^b^	0.488	Dual inhibitor of DNA topoisomerases I/II [43]	43
2	Maytansine^a^	0.554	Bind to tubulin & inhibit microtubule [41]	41
2	D-tetrandrine^b^	0.488	Calcium channel blocker & anti-inflammatory [44,45]	54

a High concentration tested: 10^–^^8.6^ M.

b High concentration tested: 10^–^^4.0^ M.

### 2.6 CellMiner Analysis of GCN2 From NCI-60 Leukemia Cell Lines

**Fig. 8:**
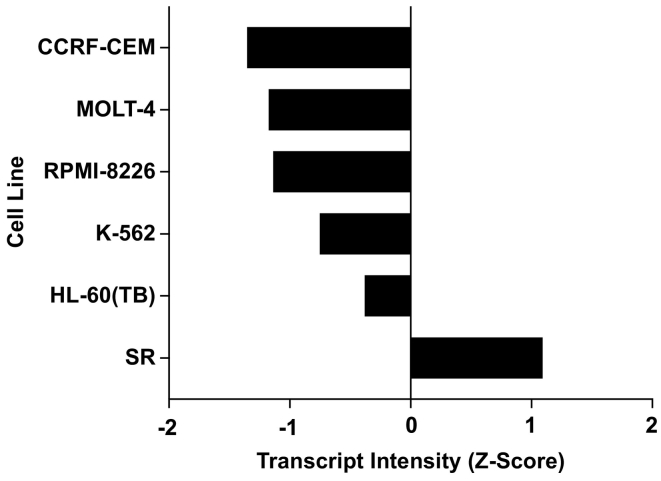
Average GCN2 Transcript Intensity (*Z*-Score) of NCI60 leukemia cell lines.

In order to determine if GCN2 expression levels were a factor in sensitivity to **1** and **2**, we utilized the CellMiner database to analyze mRNA expression data of the NCI-60 cell lines. Only SR cells had an overexpression of GCN2, and this was indeed the most sensitive cell line towards treatment with **1** and **2** (Fig. 8), although the remaining leukemia cell lines all had a negative GCN2 transcript intensity despite exhibiting sensitivity to **1** and **2**.

### 2.7 Oncomine Data Analysis

**Fig. 9:**
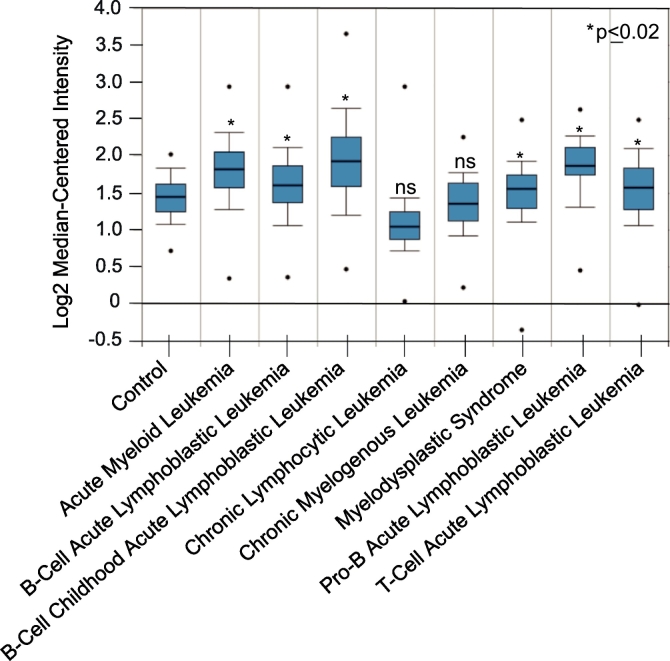
Oncomine analysis of GCN2 mRNA expression levels in leukemia relative to their normal control. Statistical significance (*, p ≤ 0.2; ns, not significant).

**Fig. 10:**
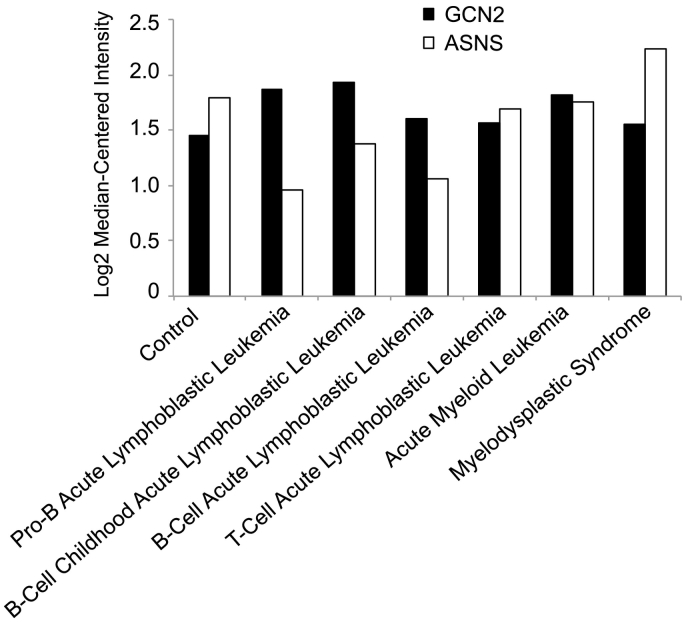
Log2 median-centered intensity of GCN2 and ASNS mRNA expression levels in leukemia relative to their normal control.

We used Oncomine genomic profiles of human cancers to test the hypothesis that GCN2 expression levels predict sensitivity to pharmacologic GCN2 inhibition. The expression levels of GCN2 were compared in normal samples (peripheral blood mononuclear cell) and different leukemia types. The results demonstrated that the GCN2 mRNA expression levels were significantly upregulated in acute myeloid leukemia, B-Cell acute lymphoblastic leukemia, B-Cell childhood acute lymphoblastic leukemia, pro-B acute lymphoblastic leukemia, and T-Cell acute lymphoblastic leukemia (Fig. 9) [42]. While a non-significant downregulation in the GCN2 mRNA expression levels was seen in both chronic lymphocytic leukemia and chronic myelogenous leukemia samples (Fig. 9). Since the GCN2-ATF4-asparagine synthetase (ASNS) pathway is known to promote tumor cell survival under nutrient deprivation [17], we hypothesized that high GCN2 and low ASNS might be a characteristic of asparaginase/GCN2-sensitive leukemia. Indeed, all B-Cell leukemia types, for which asparaginase is a licensed treatment, had an increase in GCN2 median mRNA expression levels and a simultaneous significant decrease in median mRNA ASNS expression levels (Fig. 10).

## 3 Conclusion

A large amount of convergent data suggests that GCN2 is a valid drug target in leukemias. Asparaginase treats B-ALL by amino acid deprivation, and GCN2 activation is the response to the stress of amino acid deprivation. Here, we validated a selective GCN2 chemical probe analytically, biochemically and in cells and showed that, indeed, diverse leukemia cell lines are sensitive to the probe. Cell lines with high GCN2 expression appear to be sensitive to GCN2 inhibition, as might be expected.

Analysis of our results in the context of GCN2 and ASNS mRNA expression levels from normal samples and from different leukemia types suggests interesting relationships. It stands to reason that ASNS can compensate for amino acid deprivation by replacing the missing amino acid *via* biosynthesis, and indeed, the very leukemias that show a significant high GCN2 expression and low ASNS expression are also the same B-Cell derived leukemias in which asparaginase is an effective treatment. High GCN2 expression and low ASNS expression may, therefore, predict sensitivity to a drug-like GCN2 inhibitor, but it is unclear whether low ASNS expression is necessary for sensitivity to GCN2 inhibition. Sensitivity to GCN2 inhibition may also logically correlate with sensitivity to asparaginase. The sensitivity of NK cell leukemia to asparaginase suggests that GCN2 may be a drug target in this cancer as well.

Our data suggests that other cancers may be susceptible to GCN2 inhibition. The RPMI 8226 cell line is derived from multiple myeloma and myelodysplastic syndrome, which responds to some myeloma treatments, exhibits high GCN2 expressions in the Oncomine database. We found a weaker but also uniform sensitivity of breast cancer cell lines to the validated GCN2 chemical probe, which warrants further investigation.

The consistency of the data generated with **1** and **2** suggests that GCN2 is druggable and that validated GCN2 hit inhibitors can serve as a starting point for the development of a selective *in vivo* probe for animal studies. However, the solubility of **1** and **2** is poor, which is a possible explanation for the need for micromolar concentrations needed to see the cancer cell effects. Nevertheless, these or other GCN2 selective inhibitors could be developed into lead drugs if improvements in solubility can be achieved without loss of efficacy [46]. The triazolo[4,5-*d*]pyrimidines may not be ideal drug candidates, but their inhibitory properties suggest that they should be useful as *in vitro* tool compounds when used below their maximum solubility of 0.65 μM. Most compounds analyzed in this report appear to be dual-action inhibitors of GCN2 and PKR (Table 1**, 1, 4**–**5, 8**–**9**), which may be useful in scenarios where these two kinases have synergistic effects, such as in resistance to vorinostat [47]. Thus, these compounds may serve as a blueprint for the design of certain dual action ISR inhibitors. Even though compound **6** is commercially available it has a low IC_50_ of 20.5 nM against GCN2 and it inhibits PERK by 35%. It should also be inferred that this compound will have similar solubility issues comparable to the other compounds from this series. Thus, we would caution using compound **6** at high concentrations.

The *in silico* docking studies suggest a high likelihood that **1** and **2** bind to the active site of GCN2 while adopting a similar orientation to each other and to the PERK inhibitor in PDB 4X7K. Although further SAR work and crystallographic confirmation are required to fully characterize this pocket, this model may be a good starting point to design increases in the selectivity of these compounds for GCN2. The model may also be used to diversify chemotypes *via* virtual library screening (VLS).

The NCI-60 studies for **1** and **2** revealed both compounds to have a similar cytotoxic activity. All leukemia cells tested were sensitive to both compounds, with the SR cell line exhibiting the greatest growth inhibition. In addition, both **1** and **2** have the potential to be novel from a bioactivity point of view, as similar anti-cancer activity profiles were not found in the NCI's Standard Agents database.

## 4 Methods

### 4.1 Reagents

GCN2 compounds were synthesized as previously described in the patent literature [33]. Compounds were dissolved in dimethyl sulfoxide (DMSO) to a stock concentration of 10 mM and stored at −80 °C. Compound stock solutions were thawed at room temperature before diluting into selected concentrations for use in biologic assays.

### 4.2 Cell Culture and Treatments

HEK293T cells were purchased from the American Type Culture Collection and were cultured in Dulbecco's modified Eagle's medium (DMEM; GIBCO-11995) supplemented with 10% fetal bovine serum (FBS) (Corning™ 35015CV) and antibiotics (penicillin, 10,000 UI/ml and streptomycin, 10,000 UI/ml) (Life Technologies, 15,140,122). When indicated, cells were deprived of amino acids for 4 h with DMEM (DMEM; GIBCO-21013024) containing no glutamine, no methionine, no cysteine, and no FBS. Upon starvation, cells were treated with DMSO or compound. All cell lines were maintained in humidified incubators at 37 °C under 5% CO_2_.

*Drosophila* S2 cell lines were cultured at 25 °C in Schneider's medium (Life Technologies, 21,720,024) and supplemented with 1% penicillin-streptomycin (Life Technologies, 15,140,122). For amino acid deprivation experiments, Schneider's medium lacking amino acids (USBiological Life Sciences, S0100-03) was used. Cells were treated for 6 h with 10 μM inhibitor concatenation or DMSO.

### 4.3 Kinase Assay

*In vitro* compound profiling for recombinant GCN2, PERK, HRI, PKR, or IRE1 kinases were performed at Reaction Biology Corporation (Malvern, PA) using the HotSpot kinase assay. Briefly, all compounds provided for testing were dissolved in 10 mM DMSO stock solutions. Serial dilutions were conducted by epMotion 5070 in DMSO. Specific kinase/substrate mixtures and required cofactors were prepared in reaction buffer (20 mM Hepes (pH 7.5), 10 mM MgCl_2_, 1 mM EGTA, 0.02% Brij35, 0.02 mg/ml BSA, 0.1 mM Na_3_VO_4_, 2 mM DTT, 1% DMSO). Compounds were added to the kinase reaction mixture by Acoustic technology (Echo550; nanoliter range) and incubated for 20 min at room temperature. The kinase reaction was initiated by adding ^33^P ATP to a final concentration of 10 μM for single point kinase activity reading or to the ATP Km of GCN2 for IC_50_ readings. The reaction was incubated or 2 h at room temperature and kinase activity was detected by filter-binding method. IC_50_ values and curves were generated with GraphPad Prism 7 (GraphPad software).

### 4.4 Western Blotting

Cells were lysed for 10 min on ice with RIPA lysis buffer (50 mM Tris (pH 8), 150 mM NaCl, 1% NP-40, 0.5% sodium deoxycholate, 0.1% SDS, containing Protease Inhibitor Cocktail (PIC) (ROCHE, 11836170001), 100 mM NaF, 2 mM NaVO4, 1 mM Na B-glycerophosphate). The cellular lysate was centrifuged at 16,100 ×*g* for 10 min and the supernatant was separated on 12% SDS–polyacrylamide gels and electroblotted onto nitrocellulose membrane. Primary antibodies to p-eIF2α (Ser51) (Cell Signaling #9721, 1:500 dilution), total eIF2α (Cell Signaling #9722, 1:5000 dilution), and actin (Cell Signaling #3700, 1:5000) were used. Immunoreactivity was visualized using secondary antibodies conjugated with HRP (horseradish peroxidase, Cell Signaling) or with Alexa 680 (Invitrogen, A21422) at 1:5000 dilution. IC_50_s were calculated using dose response – Inhibition nonlinear regression algorithm [[inhibitor] *vs.* response] in GraphPad Prism 7 (GraphPad Software).

### 4.5 Quantitative Real-Time RT-PCR

Total RNA was extracted with TRIzol Reagent (Invitrogen, USA). For cDNA synthesis, 200 ng of RNA was transcribed using SuperScript First-Strand Synthesis Kit (Invitrogen, USA). PCR amplification was performed for 25 cycles using *taq* polymerase (Roche) according to manufacturer's protocol. The following primer sequences were used: Thor-F 5′-GCTAAGATGTCCGCTTCACC- 3′; Thor-R 5′ CCTCC AGGAGTGGTGGAGTA-3′; Tub-F 5′- CTCAGTGCTCGATGTTGTCC-3′; Tub-R5′- CCCAAGGGAGTGTGTGAGTT-3′. Tubulin was used as a housekeeping control to normalize the amounts of cDNA between each of the samples. Results were expressed as the relative expression of mRNA levels detected in control samples and were calculated using the ΔΔCt method [48].

### 4.6 Permeability Assay (PAMPA)

GCN2 compound permeability was determined by PAMPA and was performed by Pharmaron, Inc. (Beijing, China). All compounds and control stock solutions were prepared in DMSO at a concentration of 10 mM. Testosterone and methotrexate were used as control compounds. Compound stock solutions were diluted with PBS (pH 7.4) to a final concentration of 10 μM. A 1.8% solution (w/v) of lecithin in dodecane was prepared and sonicated to ensure a complete dissolution. 5 μL of the lecithin/dodecane mixture were pipetted into each acceptor plate well (top compartment). Immediately after the application of the artificial membrane (within 10 min), 300 μL of PBS (pH 7.4) solution were added to each well of the acceptor plate. 300 μL of drug-containing solutions were added to each well of the donor plate (bottom compartment) in triplicate. The plate lid was replaced and incubated at 25 °C, 60 rpm for 16 h. After incubation, aliquots of 50 μL from each well of acceptor and donor plate were transferred into a 96-well plate and 200 μL of methanol (containing IS: 100 nM Alprazolam, 200 nM Labetalol and 2 μM Ketoprofen) was added to each well. The plate was vortexed at 750 rpm for 100 s. Samples were centrifuged at 3220*g* for 20 min. The compound concentrations were determined by LC/MS/MS. The effective permeability (Pe), in units of centimeter per second were calculated using the following equation:

$$\log\;P_{e}=\log\left\{C\times \left[-\ln\left(1-\frac{{\left[\mathrm{drug}\right]}_{\mathrm{acceptor}}}{{\left[\mathrm{drug}\right]}_{\mathrm{equilibrium}}}\right)\right]\right\}$$

### 4.7 Kinetic Solubility Assay

Compound solubility was determined in PBS at pH 7.4 by Pharmaron, Inc. (Beijing, China). Briefly, all compounds and control solutions were prepared in DMSO at concentrations of 10 mM. Diclofenac was used as a positive control. 10 μL or 30 μL of each compound was added into a 96-well plate, followed by adding 990 μL or 970 μL, respectively, of PBS at pH 7.4. A stir stick was added to each well and wells were sealed using a molded PTDE/SIL 96-Well Plate Cover. The solubility Sample plate was transferred to a Thermomixer comfort plate shaker and incubated at 25 °C for 2 h at 1100 rpm. After incubation the samples were transferred into the filter plate and filtered by Vacuum Manifold. The filtered samples were diluted with methanol to obtain 3 μM standards (STD). The samples were analyzed by LC-MS/MS against a standard of known concentration in DMSO. The solubility values of duplicate test compounds were calculated in Microsoft Excel using the following equation, where DF means dilution factor:

$$\left[\mathrm{Sample}\right]=\frac{{\mathrm{AREA}}_{\mathrm{Sample}}\times {\mathrm{INJVOL}}_{\mathrm{Std}}\times {\mathrm{DF}}_{\mathrm{Sample}}\times \left[\mathrm{STD}\right]}{{\mathrm{AREA}}_{\mathrm{Std}}\times {\mathrm{INJVOL}}_{\mathrm{Sample}}}$$

### 4.8 GCN2 3D Homology Model

3D homology models of GCN2 were generated using ICM (Molsoft LLC, La Jolla, CA) according to previously described methods [49]. Briefly, ICM-PDB homology search was used to identify template structures from the Protein Data Bank (PDB). Templates were ranked by pairwise global alignment score using the Needleman-Wunsch algorithm modified to allow zero gap-end penalties (ZEGA) [38], resolution and the status of bound ligand. Insertions/deletions were relocated outside of secondary structure elements in the alignment between the GCN2 sequence and the template sequence, except where strong local sequence signals predicted that they would perturb secondary structures [50]. The 3D model of the GCN2 kinase domain was then built onto the template using the alignment as the residue assignment guide. Side chains and loops were optimized by Biased Probability Monte Carlo (BPMC) conformational search and energy minimization to produce the final GCN2 3D homology model [51]. After the initial model was generated, several similar, structurally reasonable, alternative conformations were produced using normal mode analysis [52].

### 4.9 In Silico Docking Studies

ICM-Dock was used to screen this entire set of GCN2 models by docking known inhibitors and non-inhibitor decoys [33,37] from experimental data, the WO 2013110309 application, and the ChEMBL database according to their docking scores [33,37,39,40]. Known inhibitors were selected based on an IC_50_ of ≤100 nM, while compounds from these sources with reported IC_50_ of ≥1 mM were selected as non-inhibitors decoys. Models showing clear discrimination of true GCN2 inhibitors from decoys were retained, and used for docking of **1** and **2**. Selected full-atom 3D compound structures were flexibly docked to a grid representation of these verified rigid GCN2 model receptors within the ATP ligand-binding pocket of interest. Each docked conformation was scored based on van der Waals, solvation electrostatics, hydrophobicity and entropy energy terms, which underly and estimate binding affinities based on the changes in free energy of the unbound to bound state. All compounds were independently docked five times to account for the stochasticity nature of the algorithm and only the top scoring docking conformation of each was evaluated [40].

### 4.10 One-Dose NCI60 Human Tumor Cell Line Screen

Compounds 1 and 2 were submitted to the NCI for growth inhibition screening against its panel of 60 cancer cell lines. The NCI screening protocol has extensively been described [[53], [54], [55], [56]]. Briefly, cancerous cell lines were grown in RPMI 1640 medium containing 5% fetal bovine serum and 2 mM l-glutamine. Depending on the doubling time of each cell line, 5000–40,000 cells were seeded in 96-well plates for 24 h. Cells were then treated with compounds 1 or 2 at a concentration of 10 μM and incubated for 48 h at 37 °C in a humidified atmosphere containing 5% CO_2_. The cells were then fixed and stained with sulforhodamine B (SRB) to determine their viability. The data were analyzed using a program called COMPARE to determine the percent growth inhibition of each compound.

### 4.11 CellMiner Analysis

CellMiner database version 2.1 was used to retrieve the mRNA expression data for the NCI-60 human cancer cell lines. The database contains transcript expression values for five different microarrays of the 60 cell lines, which are normalized to generate expression profiles termed z-scores. The transcript values (z-scores) are standard deviations from the mean expression.

### 4.12 Oncomine Data Analysis

The Oncomine database (Thermo Fisher, Ann Arbor, MI) was used to analyze and visualize the mRNA expression levels of GCN2 in leukemia cells. A total of eight different leukemia types were analyzed (acute myeloid leukemia (542), T-Cell acute lymphoblastic leukemia (174), Pro-B acute lymphoblastic leukemia (70), myelodysplastic syndrome (206), chronic myelogenous leukemia (76), chronic lymphocytic leukemia (448), B-Cell childhood acute lymphoblastic leukemia (359), and B-Cell acute lymphoblastic Leukemia (147)) and analyzed against the same control (peripheral blood nononuclear cell (74)). Details of standardized normalization techniques and statistical calculations can be found on the Oncomine website (https://www.oncomine.com) and have previously been described [57]. Briefly, Oncomine uses publically available microarray datasets.

### 4.13 Software

The receptor and ligand preparations, the docking simulations, and the energy and gap evaluations were carried out with ICM 3.8-5 (Molsoft LLC, La Jolla, CA).

### 4.14 Statistical Analysis

Statistical analysis was performed with the GraphPad Prism 7.0 software. All data were depicted as the mean of individual values from at least two independent experiments.

### 4.15 Chemistry

Synthesis steps and characterization data are provided in the supplemental data.

## Appendix A Supplementary data

General Synthesis. Image 11

Compound 2 Broad Kinase Panel Screen Image 12

Percent Growth Inhibition of Compound 1 and Compound 2. Image 13

Broad Kinase Panel Screen Methods Image 14
