# Supplementary material for: Triazolo[4,5-d]pyrimidines as Validated General Control Nonderepressible 2 (GCN2) Protein Kinase Inhibitors Reduce Growth of Leukemia Cells
Source: Comput Struct Biotechnol J. 2018 Sep 28;16:350–60. doi: 10.1016/j.csbj.2018.09.003 (PMC6197744; doi:10.1016/j.csbj.2018.09.003)
Supplement: Supplementary file 1 — General Synthesis. [file mmc1.doc]

**Supporting Information**

**Methods**

**Synthesis**

The procedures were adopted from the patent application with minor modifications.

Commercially available reagents, intermediates and solvents were used as received. In general, reactions were carried out under an atmosphere of N_2_ and reaction progress was monitored by TLC (SiO_2_, Silicycle, Inc.) with UV visualization at 254 nm. Purifications were performed using flash column chromatography through SiO_2_ (230–400 mesh, Silicycle, Inc.). ^1^H NMR spectra were measured on Bruker AV-400, AV-500 or AVIII-600 High Performance Digital NMR spectrophotometers. Low-resolution mass spectra were obtained using Agilent 1100 Series Capillary LCMSD Trap XCT Spectrometer. High-resolution mass spectra were obtained using Agilent 6224 Accurate-Mass TOF/LC/MS Spectrometer. Compound names were generated using ChemDraw Professional version 16.0. In general, spectral data was in accord with reported data.

**Preparation of compounds 1–9**

Preparation of **methyl 4-(5-((1-ethyl-1*H*-pyrazol-4-yl)amino)-3*H*-[1,2,3]triazolo[4,5-*d*]pyrimidin-3-yl)benzoate** (**1**)

**Methyl 4-((2-chloro-5-nitropyrimidin-4-yl)amino)benzoate** (**12a**)

Methyl 4-aminobenzoate (**11a**, 779 mg, 5.15 mmol) was added in small portions to a solution of 2,4-dichloro-5-nitropyrimidine (**10**, 1.00 g, 5.15 mmol) in dioxane (20 mL) at 25°C. The resulting warm suspension was cautiously treated with DIPEA (985 μL, 5.66 mmol), and allowed to stir for 3 hours. The reaction mixture was poured into water, and the aqueous suspension was extracted twice with ethyl acetate. The organic layers were combined, washed with brine, dried (Na_2_SO_4_), filtered and concentrated *in vacuo* to afford the title compound as a brown solid (1.44 g, 91% unpurified yield). ^1^H NMR (400 MHz, CDCl_3_) δ 10.37 (s, 1H), 9.24 (s, 1H), 8.21 – 7.94 (m, 2H), 7.88 – 7.67 (m, 2H), 3.95 (s, 3H).

**Methyl 4-(5-chloro-3*H*-[1,2,3]triazolo[4,5-*d*]pyrimidin-3-yl)benzoate** (**13a**)

A slug of Raney Ni (~1g) was added to a solution of **12a** (1.00 g, 3.24 mmol) in THF (11 mL). The sealed flask was subjected to three cycles of evacuation and N_2_ purging. After a fourth evacuation, the mixture was allowed to stir for 1 h under 1 atm of H_2_ (balloon). The reaction was filtered through Celite® and concentrated to a black tar and used directly in the next step. NaNO_2_ (468 mg, 6.78 mmol) was added in portions to a suspension of methyl 4-((5-amino-2-chloropyrimidin-4-yl)amino)benzoate (3.24 mmol, assumed) in concentrated HCl (30 mL). The reaction mixture was allowed to stir for 2 h. The resulting free flowing tan slurry was filtered through a fritted funnel to collect a brown solid (958 mg, 76%, unpurified yield. ^1^H NMR (400 MHz, DMSO-*d_6_*) δ 9.88 (s, 1H), 8.35 – 8.27 (m, 4H), 3.92 (s, 3H).

**Methyl 4-(5-((1-ethyl-1*H*-pyrazol-4-yl)amino)-3*H*-[1,2,3]triazolo[4,5-*d*]pyrimidin-3-yl)benzoate** (**1**)

A solution of **13a** (32 mg, 0.11 mmol) and **14a** (12 mg, 0.11 mmol) in 2-methoxyethanol (1mL) was allowed to stir for 1 h at 100 ºC in a stir-bar equipped capped 1 dram vial for 3 h. The reaction mixture was concentrated *in vacuo* and the crude product was purified by flash column chromatography (5:95 MeOH:CH_2_Cl_2_). The product from the column was triturated with diethyl ether to remove trace 2-methoxyethanol. The title product was isolated as a tan solid (17.7 mg, 44%). The spectral data are consistent with the reported data.^33^ ^1^H NMR (600 MHz, DMSO-*d*_6_) δ 10.49 (s, 1H), 9.41 (s, 1H), 8.39 (d, *J* = 8.2 Hz, 2H), 8.29 (d, *J* = 8.0 Hz, 2H), 8.05 (s, 1H), 7.66 (s, 1H), 4.17 (q, *J* = 7.0 Hz, 2H), 3.93 (s, 3H), 1.42 (t, *J* = 7.1 Hz, 3H). MS ESI 365.1 [M+H]^+^, calcd for (C_17_H_16_N_8_O_2_+H)^+^ 365.1.

Preparation of **3-(4-Ethoxyphenyl)-*N*-(1-ethyl-1*H*-pyrazol-4-yl)-3*H*-[1,2,3]triazolo[4,5-*d*]pyrimidin-5-amine** (**2**)

**Methyl 4-((2-chloro-5-nitropyrimidin-4-yl)amino)benzoate** (**12b**)

4-Ethoxyaniline (**11b**, 707 mg, 5.15 mmol) was added in small portions to a solution of 2,4-dichloro-5-nitropyrimidine (**10**, 1.00 g, 5.15 mmol) in dioxane (20 mL) at 25°C. The resulting warm suspension was cautiously treated with DIPEA (985 μL, 5.66 mmol), and allowed to stir for 3 hours. The reaction mixture was poured into water, and the aqueous suspension was extracted twice with ethyl acetate. The organic layers were combined, washed with brine, dried (Na_2_SO_4_), filtered and concentrated *in vacuo*. The crude solid was purified using flash column chromatography (1:4 EtOAc:hexanes). The fractions containing the pure product were concentrated *in vacuo* to afford the title compound as a red solid (1.12 g, 74%). ^1^H NMR (400 MHz, CDCl_3_) δ 10.04 (s, 1H), 9.09 (s, 1H), 7.55 – 7.36 (m, 2H), 7.01 – 6.80 (m, 2H), 4.01 (q, *J* = 7.0 Hz, 2H), 1.38 (t, *J* = 7.0 Hz, 3H).

**Methyl 4-(5-chloro-3*H*-[1,2,3]triazolo[4,5-*d*]pyrimidin-3-yl)benzoate** (**13b**)

A slug of Raney Ni (~1g) was added to a solution of **12b** (1.00 g, 3.39 mmol) in THF (11 mL). The sealed flask was subjected to three cycles of evacuation and N_2_ purging. After a fourth evacuation, the mixture was allowed to stir for 1 h under 1 atm of H_2_ (balloon). The reaction was filtered through Celite® and concentrated to a black tar and used directly in the next step. NaNO_2_ (468 mg, 6.78 mmol) was added in portions to a suspension of 2-chloro-*N*^4^-(4-ethoxyphenyl)pyrimidine-2,5-diamine (3.39 mmol, assumed) in concentrated HCl (30 mL). The reaction mixture was allowed to stir for 2 h. The resulting free flowing tan slurry was filtered through a fritted funnel to collect a light brown solid, which was used without further purification (3.40 g, 100% unpurified yield). ^1^H NMR (400 MHz, DMSO-*d_6_*) δ 9.83 (s, 1H), 7.91 (d, *J* = 9.0 Hz, 2H), 7.24 (d, *J* = 9.1 Hz, 2H), 4.15 (q, *J* = 7.0 Hz, 2H), 1.38 (t, *J* = 7.0 Hz, 3H).

**3-(4-Ethoxyphenyl)-*N*-(1-ethyl-1*H*-pyrazol-4-yl)-3*H*-[1,2,3]triazolo[4,5-*d*]pyrimidin-5-amine** (**2**)

A solution of **13b** (32 mg, 0.11 mmol) and **14a** (12 mg, 0.11 mmol) in 2-methoxyethanol (1mL) was allowed to stir for 1 h at 100 ºC in a stir-bar equipped capped 1 dram vial for 3 h. The reaction mixture was concentrated *in vacuo* and the crude product was purified by flash column chromatography (5:95 MeOH:CH_2_Cl_2_). The product from the column was triturated with diethyl ether to remove trace 2-methoxyethanol. The title product was isolated as a tan solid (18.5 mg, 48%). The spectral data are consistent with the reported data.^33^ ^1^H NMR (600 MHz, DMSO-*d*_6_) δ 10.37 (s, 1H), 9.36 (s, 1H), 8.00 (s, 3H), 7.62 (s, 1H), 7.23 (d, *J* = 8.3 Hz, 2H), 4.13 (dq, *J* = 14.6, 6.9 Hz, 4H), 1.39 (t, *J* = 5.1 Hz, 6H). MS ESI 373.2 [M+Na]^+^, calcd for (C_17_H_18_N_8_O+Na)^+^ 373.2.

Preparation of **3-(4-Ethoxyphenyl)-*N*-(1-(tetrahydro-2*H*-pyran-4-yl)-1*H*-pyrazol-4-yl)-3*H*-[1,2,3]triazolo[4,5-*d*]pyrimidin-5-amine** (**3**)

A solution of **13b** (30 mg, 0.11 mmol) and **14b** (18 mg, 0.11 mmol) in 2-methoxyethanol (1mL) was allowed to stir for 1 h at 100 ºC in a stir-bar equipped capped 1 dram vial for 3 h. The reaction mixture was concentrated *in vacuo* and the crude product was purified by flash column chromatography (5:95 MeOH:CH_2_Cl_2_). The product from the column was triturated with diethyl ether to remove trace 2-methoxyethanol. Title compound isolated as a tan solid (21.4 mg, 48%). The spectral data are consistent with the reported data.^33^ ^1^H NMR (600 MHz, DMSO-*d*_6_) δ 10.41 (s, 1H), 9.38 (s, 1H), 8.13 (s, 1H), 8.00 (d, *J* = 8.4 Hz, 2H), 7.63 (s, 1H), 7.23 (d, *J* = 8.6 Hz, 2H), 4.43 – 4.35 (m, 1H), 4.16 (q*, J* = 6.8 Hz, 2H), 3.97 (d, *J* = 13.7 Hz, 2H), 3.50 (t, *J* = 11.4 Hz, 2H), 2.03 (d, *J* = 12.9 Hz, 2H), 1.96 – 1.82 (m, 2H), 1.40 (t, *J* = 6.9 Hz, 3H). MS ESI 429.2 [M+Na]^+^, calcd for (C_20_H_22_N_8_O_2_+Na)^+^ 429.2.

Preparation of **3-(4-Chloro-3-fluorophenyl)-*N*-(1-(tetrahydro-2*H*-pyran-4-yl)-1*H*-pyrazol-4-yl)-3*H*-[1,2,3]triazolo[4,5-*d*]pyrimidin-5-amine** (**4**)

**2-Chloro-*N*-(4-chloro-3-fluorophenyl)-5-nitropyrimidin-4-amine** (**12c**)

4-Chloro-3-fluoroaniline (**11c**, 750 mg, 5.15 mmol) was added in small portions to a solution of 2,4-dichloro-5-nitropyrimidine (**10**, 1.00 g, 5.15 mmol) in dioxane (20 mL) at 25°C. The resulting warm suspension was cautiously treated with DIPEA (985 μL, 5.66 mmol), and allowed to stir for 3 hours. The reaction mixture was poured into water, and the aqueous suspension was extracted twice with ethyl acetate. The organic layers were combined, washed with brine, dried (Na_2_SO_4_), filtered and concentrated *in vacuo* to afford after column chromatography (1:4 EtOAc:hexanes) the desired product as a brown solid (1.12 g, 72%). ^1^H NMR (400 MHz, CDCl_3_) δ 10.25 (s, 1H), 9.23 (d, *J* = 1.5 Hz, 1H), 7.73 (dd, *J* = 10.4, 2.5 Hz, 1H), 7.51 – 7.44 (m, 1H), 7.31 (ddd, *J* = 8.8, 2.5, 1.2 Hz, 1H).

**Methyl 4-(5-chloro-3*H*-[1,2,3]triazolo[4,5-*d*]pyrimidin-3-yl)benzoate** (**13c**)

A slug of Raney Ni (~1g) was added to a solution of **12c** (1.00 g, 3.30 mmol) in THF (11 mL). The sealed flask was subjected to three cycles of evacuation and N_2_ purging. After a fourth evacuation, the mixture was allowed to stir for 1 h under 1 atm of H_2_ (balloon). The reaction was filtered through Celite® and concentrated to a black tar and used directly in the next step. NaNO_2_ (468 mg, 6.78 mmol) was added in portions to a suspension of 2-chloro-*N*^4^-(4-chloro-3-fluorophenyl)pyrimidine-4,5-diamine (3.30 mmol, assumed) in concentrated HCl (30 mL). The reaction mixture was allowed to stir for 2 h. The resulting free flowing tan slurry was filtered through a fritted funnel to collect a tan solid (626 mg, 49% unpurified yield). ^1^H NMR (400 MHz, DMSO-*d_6_*) δ 9.88 (s, 1H), 8.22 (dd, *J* = 10.0, 2.2 Hz, 1H), 8.08 – 8.03 (m, 1H), 8.03 – 7.97 (m, 1H).

**3-(4-Chloro-3-fluorophenyl)-*N*-(1-(tetrahydro-2*H*-pyran-4-yl)-1*H*-pyrazol-4-yl)-3*H*-[1,2,3]triazolo[4,5-d]pyrimidin-5-amine** (**4**)

A solution of **13c** (31 mg, 0.11 mmol) and **14b** (18 mg, 0.11 mmol) in 2-methoxyethanol (1mL) was allowed to stir for 1 h at 100 ºC in a stir-bar equipped capped 1 dram vial for 3 h. The reaction mixture was concentrated *in vacuo* and the crude product was purified by flash column chromatography (5:95 MeOH:CH_2_Cl_2_). The product from the column was triturated with diethyl ether to remove trace 2-methoxyethanol. Title compound isolated as a tan solid (14.2mg, 31%): ^1^H NMR (600 MHz, DMSO-*d*_6_) δ 10.50 (s, 1H), 9.41 (s, 1H), 8.32 (d, *J* = 11.6 Hz, 1H), 8.12 (s, 1H), 8.07 (d, *J* = 8.7 Hz, 1H), 7.94 (t, *J* = 8.4 Hz, 1H), 7.65 (s, 1H), 4.40 (ddd, *J* = 15.6, 11.3, 4.3 Hz, 1H), 3.98 (dd, *J* = 10.9, 3.3 Hz, 2H), 3.50 (t, *J* = 11.2 Hz, 2H), 2.04 (d, *J* = 11.9 Hz, 2H), 1.99 – 1.88 (m, 2H). HRMS (APCI) *m/z* calcd for (C_18_H_16_ClFN_8_O) 414.1120, found 414.1112.

Preparation of **Methyl 4-(5-((1-(tetrahydro-2*H*-pyran-4-yl)-1*H*-pyrazol-4-yl)amino)-3*H*-[1,2,3]triazolo[4,5-*d*]pyrimidin-3-yl)benzoate** (**5**)

A solution of **13a** (32 mg, 0.11 mmol) and **14b** (18 mg, 0.11 mmol) in 2-methoxyethanol (1mL) was allowed to stir for 1 h at 100 ºC in a stir-bar equipped capped 1 dram vial for 3 h. The reaction mixture was concentrated *in vacuo* and the crude product was purified by flash column chromatography (5:95 MeOH:CH_2_Cl_2_). The product from the column was triturated with diethyl ether to remove trace 2-methoxyethanol. Title compound isolated as a tan solid (16.5 mg, 36%). The spectral data are consistent with the reported data.^33^ ^1^H NMR (600 MHz, DMSO-*d*_6_) δ 10.52 (s, 1H), 9.42 (s, 1H), 8.38 (d, *J* = 8.5 Hz, 2H), 8.28 (d, *J* = 8.4 Hz, 2H), 8.18 (s, 1H), 7.66 (s, 1H), 4.47 – 4.37 (m, 1H), 4.04 – 3.96 (m, 2H), 3.93 (s, 3H), 3.52 (t, *J* = 11.3 Hz, 2H), 2.08 (d, *J* = 11.0 Hz, 2H), 1.93 (td, *J* = 11.7, 5.6 Hz, 2H). MS ESI 419.4 [M-H]^-^, calcd for (C_20_H_20_N_8_O_3_-H)^-^ 419.2.

Preparation of **3-(1*H*-Indazol-6-yl)-N-(1-(tetrahydro-2*H*-pyran-4-yl)-1*H*-pyrazol-4-yl)-3*H*-[1,2,3]triazolo[4,5-*d*]pyrimidin-5-amine** (**6**)

***N*-(2-chloro-5-nitropyrimidin-4-yl)-1*H*-indazol-6-amine** (**12d**)

6-Aminoindazole (**11d**, 686 mg, 5.15 mmol) was added in small portions to a solution of 2,4-dichloro-5-nitropyrimidine (**10**, 1.00 g, 5.15 mmol) in dioxane (20 mL) at 25°C. The resulting warm suspension was cautiously treated with DIPEA (985 μL, 5.66 mmol), and allowed to stir for 3 hours. The reaction mixture was poured into water, and the aqueous suspension was extracted twice with ethyl acetate. The organic layers were combined, washed with brine, dried (Na_2_SO_4_), filtered and concentrated *in vacuo* to afford the title compound as a tan solid (1.52 g, 100% unpurified yield). ^1^H NMR (400 MHz, DMSO-*d_6_*) δ 13.17 (s, 1H), 10.55 (s, 1H), 9.17 (s, 1H), 8.09 (s, 1H), 7.80 (d, *J* = 13.5 Hz, 2H), 7.22 (dd, *J* = 8.6, 1.7 Hz, 1H).

**Methyl 4-(5-chloro-3*H*-[1,2,3]triazolo[4,5-*d*]pyrimidin-3-yl)benzoate** (**13d**)

A slug of Raney Ni (~1g) was added to a solution of **12d** (1.00 g, 3.44 mmol) in THF (11 mL). The sealed flask was subjected to three cycles of evacuation and N_2_ purging. After a fourth evacuation, the mixture was allowed to stir for 1 h under 1 atm of H_2_ (balloon). The reaction was filtered through Celite® and concentrated to a black tar and used directly in the next step. NaNO_2_ (468 mg, 6.78 mmol) was added in portions to a suspension of 2-chloro-*N*^4^-(1*H*-indazol-6-yl)pyrimidine-4,5-diamine (3.44 mmol, assumed) in concentrated HCl (30 mL). The reaction mixture was allowed to stir for 2 h. The resulting free flowing tan slurry was filtered through a fritted funnel to collect a brown solid. The crude title compound was purified by column chromatography (5:95 MeOH:CH_2_Cl­_2_) to afford the desired product **13d** as a tan solid (103 mg, 11%). ^1^H NMR (400 MHz, DMSO-*d_6_*) δ 13.49 (s, 1H), 9.87 (s, 1H), 8.29 (d, *J* = 15.3 Hz, 2H), 8.11 (d, *J* = 8.6 Hz, 1H), 7.84 (dd, *J* = 8.7, 1.7 Hz, 1H).

**3-(1*H*-Indazol-6-yl)-N-(1-(tetrahydro-2*H*-pyran-4-yl)-1*H*-pyrazol-4-yl)-3*H*-[1,2,3]triazolo[4,5-*d*]pyrimidin-5-amine** (**6**)

A solution of **13d** (30 mg, 0.11 mmol) and **14b** (18 mg, 0.11 mmol) in 2-methoxyethanol (1mL) was allowed to stir for 1 h at 100 ºC in a stir-bar equipped capped 1 dram vial for 3 h. The reaction mixture was concentrated *in vacuo* and the crude product was purified by flash column chromatography (5:95 MeOH:CH_2_Cl_2_).Title compound isolated as an off-white solid (9.6 mg, 48%) after triturating the purified material in methanol. The spectral data are consistent with the reported data.^33^ ^1^H NMR (600 MHz, DMSO-*d*_6_) δ 13.43 (s, 1H), 10.46 (s, 1H), 9.42 (s, 1H), 8.26 - 8.22 (m, 2H), 8.13 (s, 1H), 8.09 (d, *J* = 8.3 Hz, 1H), 7.89 (d, *J* = 8.5 Hz, 1H), 7.67 (s, 1H), 4.35 (t, *J* = 10.4 Hz, 1H), 3.92 (d, *J* = 11.3 Hz, 2H), 3.45 (t, *J* = 11.5 Hz, 2H), 2.00 (d, *J* = 11.7 Hz, 2H), 1.83 (d, *J* = 7.2 Hz, 2H). MS ESI 401.3 [M-H]^-^, calcd for (C_19_H_10_N_10_O-H)^-^ 401.2.

Preparation of ***trans*-4-(4-((3-(4-Ethoxyphenyl)-3*H*-[1,2,3]triazolo[4,5-*d*]pyrimidin-5-yl)amino)-1*H*-pyrazol-1-yl)cyclohexan-1-ol** (**7**)

A solution of **13b** (30 mg, 0.11 mmol) and **14c** (20 mg, 0.11 mmol) in 2-methoxyethanol (1mL) was allowed to stir for 1 h at 100 ºC in a stir-bar equipped capped 1 dram vial for 3 h. The reaction mixture was concentrated *in vacuo* and the crude product was purified by flash column chromatography (5:95 MeOH:CH_2_Cl_2_). The product from the column was triturated with diethyl ether to remove trace 2-methoxyethanol. Title compound isolated as a light brown solid (28.0 mg, 40%): ^1^H NMR (400 MHz, CDCl_3_) δ 9.19 (s, 1H), 8.05 (d, J = 8.9 Hz, 2H), 8.00 (s, 1H), 7.65 (s, 1H), 7.40 (s, 1H), 7.09 (d, J = 9.0 Hz, 2H), 4.14 (q, J = 7.0 Hz, 3H), 3.78 (tt, J = 10.5, 4.1 Hz, 1H), 2.27 (d, J = 14.0 Hz, 2H), 2.18 (d, J = 14.1 Hz, 2H), 1.88 (q, J = 15.4, 14.3 Hz, 2H), 1.50 (m, J = 7.0 Hz, 5H). HRMS (APCI) *m/z* calcd for (C_21_H_24_N_8_O_2_) 420.2022, found 420.2014.

Preparation of ***trans*-4-(4-((3-(4-chloro-3-fluorophenyl)-3*H*-[1,2,3]triazolo[4,5-*d*]pyrimidin-5-yl)amino)-1*H*-pyrazol-1-yl)cyclohexan-1-ol** (**8**)

A solution of **13c** (31 mg, 0.11 mmol) and **14c** (20 mg, 0.11 mmol) in 2-methoxyethanol (1mL) was allowed to stir for 1 h at 100 ºC in a stir-bar equipped capped 1 dram vial for 3 h. The reaction mixture was concentrated *in vacuo* and the crude product was purified by flash column chromatography (5:95 MeOH:CH_2_Cl_2_). The product from the column was triturated with diethyl ether to remove trace 2-methoxyethanol. Title compound (19.2 mg, 27%): ^1^H NMR (400 MHz, DMSO-*d_6_*) δ 10.48 (s, 1H), 9.39 (s, 1H), 8.32 (d, *J* = 10.0 Hz, 1H), 8.11 – 8.01 (m, 2H), 7.92 (t, *J* = 8.4 Hz, 1H), 7.58 (s, 1H), 4.69 (d*, J* = 4.0 Hz, 1H), 4.13 – 4.05 (m, 1H), 3.50 (ddd, *J* = 15.0, 9.9, 5.6 Hz, 1H), 2.05 (d, *J* = 13.2 Hz, 2H), 1.94 (d, *J* = 11.8 Hz, 2H), 1.74 (q, *J* = 13.4, 11.3 Hz, 2H), 1.43 – 1.31 (m, 2H). HRMS (APCI) *m/z* calcd for (C_19_H_18_ClFN_8_O) 428.1276 found, 428.1270.

Preparation of **methyl 4-(5-((1-(*trans*-4-hydroxycyclohexyl)-1*H*-pyrazol-4-yl)amino)-3*H*-[1,2,3]triazolo[4,5-*d*]pyrimidin-3-yl)benzoate** (**9**)

A solution of **13a** (32 mg, 0.11 mmol) and **14c** (20 mg, 0.11 mmol) in 2-methoxyethanol (1mL) was allowed to stir for 1 h at 100 ºC in a stir-bar equipped capped 1 dram vial for 3 h. The reaction mixture was concentrated *in vacuo* and the crude product was purified by flash column chromatography (5:95 MeOH:CH_2_Cl_2_). The product from the column was triturated with diethyl ether to remove trace 2-methoxyethanol. Title compound isolated as a tan solid (24.0 mg, 33%): ^1^H NMR (400 MHz, CDCl_3_) δ 9.21 (s, 1H), 8.40 (d, *J* = 8.8 Hz, 2H), 8.29 (d, *J* = 8.7 Hz, 2H), 8.03 (s, 1H), 7.66 (s, 1H), 7.46 (s, 1H), 4.23 – 4.12 (m, 1H), 4.00 (s, 3H), 3.87 – 3.74 (m, 1H), 2.30 (d, *J* = 12.0 Hz, 2H), 2.20 (d, *J* = 11.3 Hz, 2H), 1.97 – 1.81 (m, 2H), 1.62 – 1.53 (m, 2H, obscured). HRMS (APCI) *m/z* calcd for (C_21_H_22_N_8_O_3_) 434.1815, found 434.1808.

**Preparation of intermediates 14b and 14c (14a is commercially available):**

**1-(Tetrahydro-2*H*-pyran-4-yl)-1*H*-pyrazol-4-amine** (**14b**)

**4-Nitro-1-(tetrahydro-2*H*-pyran-4-yl)-1*H*-pyrazole**

Di-*t*-butyl azodicarboxylate (981 mg, 4.86 mmol) was added, dropwise to a stirring solution of 4-nitro-1*H*-pyrazole (500 mg, 4.42 mmol), tetrahydro-4-pyranol (590 uL, 6.19 mmol), and triphenylphosphine (1.27 g, 4.86 mmol) in THF (15 mL). The reaction was allowed to stir overnight at room temperature under N_2_. The crude reaction mixture was poured into water and extracted with ethyl acetate. The combined organic layers were washed with water followed by brine, dried (Na_2_SO_4_), filtered and concentrated *in vacuo* to a crude oil. Purification by flash column chromatography (35:75 EtOAc:hexanes – EtOAc gradient elution) afforded the desired product as a white solid (760 mg, 81%). ^1^H NMR (400 MHz, CDCl_3_) δ 8.19 (s, 1H), 8.10 (s, 1H), 4.39 (ddd, *J* = 15.8, 11.4, 4.4 Hz, 1H), 4.15 (dd, *J* = 11.9, 3.6 Hz, 2H), 3.56 (td, *J* = 11.9, 2.3 Hz, 2H), 2.21 – 2.00 (m, 4H).

**1-(Tetrahydro-2*H*-pyran-4-yl)-1*H*-pyrazol-4-amine** (**14b**)^1^

A mixture of 4-Nitro-1-(tetrahydro-*2H*-pyran-4-yl)-1H-pyrazole (760 mg, 3.60 mmol) and 10% palladium on carbon (867 mg, 0.36 mmol) in MeOH (25 mL) was subjected to 3 cycles of evacuation and N_2_ purging. After a fourth evacuation, the mixture was allowed to stir at 25°C under 1 atm of H_2_ (balloon). After 1h, the mixture was filtered through Celite® and concentrated to a crude solid which was purified by flash column chromatography (1:9 MeOH:CH_2_Cl_2_) to afford the desired product as 530 mg of a white solid (78%.). The spectral data are consistent with the reported data.^38^ ^1^H NMR (400 MHz, CDCl_3_) δ 7.17 (s, 1H), 7.06 (s, 1H), 4.22 (tt, *J* = 10.1, 5.0 Hz, 1H), 4.15 – 4.04 (m, 2H), 3.52 (td, *J* = 11.6, 3.0 Hz, 2H), 2.84 (s, 2H), 2.07 – 1.93 (m, 4H).

**(*trans*)-4-(4-Amino-1*H*-pyrazol-1-yl)cyclohexan-1-ol** (**14c**)^2^

**4-Nitro-1-(1,4-dioxaspiro[4.5]decan-8-yl)-1*H*-pyrazole**

Prepared using 4-nitro-1*H*-pyrazole (325 mg, 2.87 mmol) and 1,4-dioxaspiro[4.5]decan-8-ol (500 mg, 3.16 mmol) and the same procedure for 4-Nitro-1-(tetrahydro-2*H*-pyran-4-yl)-1*H*-pyrazole (4.8.4.2.1) Purification by flash column chromatography (35:75 EtOAc:hexanes – EtOAc gradient elution) afforded the desired product as a white solid (470 mg, 65%). The spectral data are consistent with the reported data.^2^ ^1^H NMR (400 MHz, CDCl_3_) δ 8.27 (s, 1H), 8.19 (s, 1H), 8.08 (s, 1H), 4.98 (dt, *J* = 12.5, 6.3 Hz, 1H), 4.25 (ddd, *J* = 15.5, 11.4, 4.1 Hz, 1H), 3.99 (s, 4H), 2.21 (dd, *J* = 13.1, 3.9 Hz, 2H), 2.09 (qd, *J* = 12.8, 4.7 Hz, 3H), 1.91 (d, *J* = 12.5 Hz, 2H), 1.75 (td, *J* = 13.4, 4.4 Hz, 2H).

**4-(4-Nitro-1*H*-pyrazol-1-yl)cyclohexan-1-one**

4-Nitro-1-(1,4-dioxaspiro[4.5]decan-8-yl)-1*H*-pyrazole (470mg, 1.86 mmol) was dissolved in 1:1 acetone;water (4mL), treated with pyridinium *p*-toluenesulfonate (936 mg, 3.72 mmol) and heated to 65 °C. After 5 hours, the mixture was concentrated *in vacuo* to remove the acetone, diluted with water and extracted twice with ethyl acetate. The combined organic layers were dried (Na_2_SO_4_), filtered, and concentrated *in vacuo*. Purification by flash column chromatography (5:95 MeOH:CH_2_Cl_2_) afforded the title compound as a white solid (276 mg, 71%). The spectral data are consistent with the reported data.^2^ ^1^H NMR (400 MHz, CDCl_3_) δ 8.23 (s, 1H), 8.11 (s, 1H), 4.65 (tt, *J* = 10.2, 3.9 Hz, 1H), 2.68 – 2.46 (m, 6H), 2.33 (ddt, *J* = 17.6, 13.0, 6.6 Hz, 2H).

**(*trans*)-4-(4-Nitro-1*H*-pyrazol-1-yl)cyclohexan-1-ol**

Sodium borohydride (25mg, 0.66mmol) was added to a solution of 4-(4-nitro-1*H*-pyrazol-1-yl)cyclohexan-1-one (276mg, 1.32mmol) in ethanol (10mL). After 2 hours. The reaction was poured into water and extracted with ethyl acetate. The combined organic layers were washed with brine, dried over Na_2_SO_4_, filtered, concentrated and purified by flash column chromatography (40% EtOAC in pet. ether). The desired product is the 2^nd^ eluting product and was isolated as a white solid (150 mg, 53%). The spectral data are consistent with the reported data.^2^ ^1^H NMR (400 MHz, CDCl_3_) δ 8.15 (s, 1H), 8.08 (s, 1H), 4.16 (tt, *J* = 11.7, 3.9 Hz, 1H), 3.77 (ddt, *J* = 10.7, 8.5, 4.3 Hz, 1H), 2.31 – 2.21 (m, 2H), 2.17 (dd, *J* = 14.0, 3.5 Hz, 2H), 1.87 (qd, *J* = 13.1, 3.5 Hz, 2H), 1.55 – 1.45 (m, 2H).

**(*trans*)-4-(4-Amino-1*H*-pyrazol-1-yl)cyclohexan-1-ol** (**14c**)

(*trans*)-4-(4-Nitro-1*H*-pyrazol-1-yl)cyclohexan-1-ol was hydrogenated over 10% palladium on carbon as described for **6b**. After flash column chromatography (1:9 MeOH:CH_2_Cl_2_) the desired product was isolated as a white solid (120 mg, 64%.). The spectral data are consistent with the reported data.^2^ ^1^H NMR (400 MHz, Methanol-*d_4_*) δ 7.25 (d, *J* = 0.7 Hz, 1H), 7.15 (d, *J* = 0.7 Hz, 1H), 4.02 (tt, *J* = 12.1, 3.5 Hz, 1H), 3.64 (tt, *J* = 11.0, 3.8 Hz, 1H), 2.12 – 2.01 (m, 4H), 1.82 (qd, *J* = 12.3, 2.5 Hz, 2H), 1.55 – 1.40 (m, 2H).

References:

1. Boyer, S. J.; Gao, D.; Guo, X.; Kirrane, T.; Sarko, C. R.; Snow, R. J.; Soleymanzadeh, F.; Zhang, Y. Preparation of heteroaryl oxotetrahydrodiazepino indole carboxamide derivatives and analogs for use as RSK inhibitors. June 16, 2011.

2. Jin, M.; Mulvihill, M. J.; Steinig, A. G.; Wang, J., Preparation of fused bicyclic heterocycles as kinase inhibitors. WO 2012158658, November 22, 2012.
