# Supplementary material for: Triazolo[4,5-d]pyrimidines as Validated General Control Nonderepressible 2 (GCN2) Protein Kinase Inhibitors Reduce Growth of Leukemia Cells
Source: Comput Struct Biotechnol J. 2018 Sep 28;16:350–60. doi: 10.1016/j.csbj.2018.09.003 (PMC6197744; doi:10.1016/j.csbj.2018.09.003)
Supplement: Supplementary file 2 — Compound 2 Broad Kinase Panel Screen [file mmc2.pdf]

Kinase	Family	% Activity Remaining<br>Compound 2 (0.5 uM)
AAK1	Other	73.5
ABL1	TK	88.9
ACVR2A/ACVR2	TKL	100.0
AKT1	AGC	98.4
AKT2	AGC	96.8
AKT3	AGC	100.0
AMPK-a1/AMPK	CAMK	100.0
AURKA	Other	97.1
AURKB	Other	94.7
AURKC	Other	98.7
CAMK1D	CAMK	92.9
CAMK2D	CAMK	89.9
CDK5	CMGC	94.4
CLK2	CMGC	93.0
DAPK3	CAMK	97.6
DDR1	TK	93.1
EPHA5	TK	97.8
EPHB2	TK	97.7
FLT1/VEGFR1	TK	51.7
FLT3	TK	100.0
HCK	TK	96.0
IGF1R	TK	100.0
ITK	TK	89.9
KIT	TK	88.8
MAP3K10/MLK2	TKL	96.7
MAP3K11/MLK3	TKL	100.0
MAPK14/p38a	CMGC	100.0
MARK1	CAMK	100.0
MARK2	CAMK	99.3
MSK2/RPS6KA4	AGC	100.0
MST2	STE	100.0
MUSK	TK	100.0
PDGFRB	TK	84.4
PDK1/PDPK1	AGC	96.2
PIM1	CAMK	93.4
PKA/PRKACA	AGC	100.0
PKCe/PRKCE	AGC	100.0
PKG1/PRKG1	AGC	96.0
PKX/PRKX	AGC	97.7
PLK4	Other	100.0
PTK2B/PYK2	TK	96.0
RSK2/RPS6KA3	AGC	89.9
SLK	STE	98.7
SIK1/SNF1LK	CAMK	99.9
SIK2	CAMK	100.0
SRC	TK	99.1
TNK1	TK	100.0
VEGFR2/KDR/FLK1	TK	77.1
YANK2	AGC	100.0
YSK1	STE	93.6

Supplementary Material S2. Compound **2** broad kinase panel screen.
