# Supplementary material for: Triazolo[4,5-d]pyrimidines as Validated General Control Nonderepressible 2 (GCN2) Protein Kinase Inhibitors Reduce Growth of Leukemia Cells
Source: Comput Struct Biotechnol J. 2018 Sep 28;16:350–60. doi: 10.1016/j.csbj.2018.09.003 (PMC6197744; doi:10.1016/j.csbj.2018.09.003)
Supplement: Supplementary file 3 — Percent Growth Inhibition of Compound 1 and Compound 2. [file mmc3.pdf]

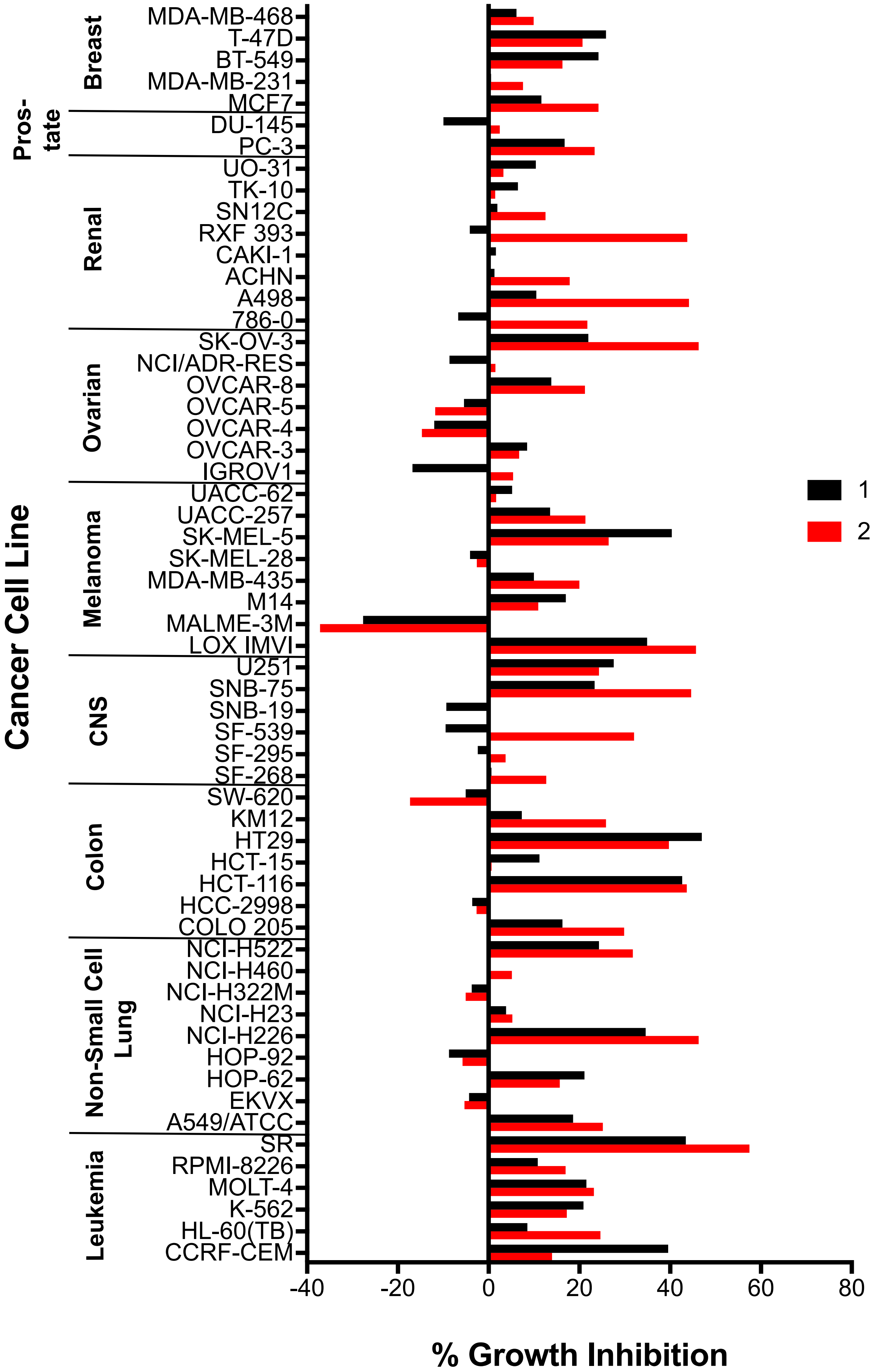

Supplementary Material S3. Percent growth inhibition by cancer type of **1** and **2** from the NCI-60 one-dose screen.
