# Supplementary material for: Triazolo[4,5-d]pyrimidines as Validated General Control Nonderepressible 2 (GCN2) Protein Kinase Inhibitors Reduce Growth of Leukemia Cells
Source: Comput Struct Biotechnol J. 2018 Sep 28;16:350–60. doi: 10.1016/j.csbj.2018.09.003 (PMC6197744; doi:10.1016/j.csbj.2018.09.003)
Supplement: Supplementary file 4 — Broad Kinase Panel Screen Methods [file mmc4.docx]

**Broad Kinase Panel Screen**

Compound **2** was screened against a standard panel of 50 protein kinases by Luceome Biotechnologies (Tucson, AZ). In brief, Compound **2** was dissolved and diluted in DMSO to a concentration of 12.5 μM from a 10 mM stock solution. As a control, compound **2** was evaluated for false positive against split-luciferase. For kinase assays, each C-terminal half of firefly luciferase linked kinase (Cfluc-Kinase) was translated along with the coiled coil Fos attached to the N-terminal fragment (Nfluc) (Fos-Nfluc) using rabbit reticulocyte lysate at 30 °C for 90 min. 24 μL aliquot of this lysate containing 1 μL of either DMSO or Compound **2** (at a final concentration 0.5 μM) was incubated for 30 minutes at room temperature followed by 1 hour incubation in the presence of a kinase specific probe. Finally, 80 μL of luciferin assay reagent was added and luminescence was immediately measured on a luminometer. Compound **2** was screened in duplicate against each kinase.

The % Inhibition and % Activity Remaining was calculated using the following equations:
